# High-flow nasal cannula therapy with sequential noninvasive ventilation versus noninvasive ventilation alone as the initial ventilatory strategy in acute COPD exacerbations: study protocol for a randomized controlled trial

**DOI:** 10.1186/s13063-022-06963-w

**Published:** 2022-12-29

**Authors:** Shuai Liu, Joseph Harold Walline, Huadong Zhu, Yan Li, Chunting Wang, Jihai Liu

**Affiliations:** 1 Emergency Department, State Key Laboratory of Complex Severe and Rare Diseases, Peking Union Medical College Hospital, Chinese Academy of Medical Science and Peking Union Medical College, Beijing, 100730 China; 2 Centre for the Humanities and Medicine, The University of Hong Kong, Hong Kong, China

**Keywords:** Chronic obstructive pulmonary disease, High-flow nasal cannula, Hospital emergency service, Intensive care units, Noninvasive ventilation, Respiratory insufficiency

## Abstract

**Background:**

Noninvasive ventilation (NIV) is the recommended mode of ventilation used in acute respiratory failure secondary to an acute exacerbation of chronic obstructive pulmonary disease (AECOPD). Recent data has shown that high-flow nasal cannula (HFNC) treatment can be an alternative for patients with hypercapnic respiratory failure. The purpose of this study is to evaluate HFNC with sequential NIV versus NIV alone as the initial ventilatory strategy in AECOPD.

**Methods:**

This investigator-initiated, unblinded, single center, randomized controlled trial will be conducted in the emergency department, emergency intensive care unit, or respiratory intensive care unit of a tertiary-care urban teaching hospital. A total of 66 patients will be enrolled and randomized into the intervention group (HFNC with sequential NIV) or the control group (NIV group). The primary endpoint will be the mean difference in PaCO_2_ from baseline to 24 h after randomization. Secondary endpoints include the mean difference in PaCO_2_ from baseline to 6, 12, and 18 h, as well as the dyspnea score, overall discomfort score, rate of treatment failure, respiratory rate, rate of endotracheal intubation, length of hospital stay, and mortality.

**Discussion:**

Taking the advantages of both HFNC and NIV on AECOPD patients into account, we designed this clinical trial to investigate the combination of these ventilatory strategies. This trial will help us understand how HFNC with sequential NIV compares to NIV alone in treating AECOPD patients.

**Trial registration:**

ChiCTR2100054809.

## Introduction

### Background and rationale {6a}

Chronic obstructive pulmonary disease (COPD) is a common disease characterized by persistent respiratory symptoms and airflow limitation due to airway or alveolar abnormalities and is often caused by exposure to noxious particles or gasses [1]. The most severe exacerbation episodes may be associated with acute respiratory failure (ARF) deeming conventional oxygen therapy (COT) necessary, which is characterized by an acute worsening of respiratory symptoms. Patients with an AECOPD often present in ARF to the emergency department (ED), with or without carbon dioxide (CO_2_) retention and respiratory acidosis [1].

Noninvasive ventilation (NIV) is one of the most important treatments for patients with AECOPD [2]. The Global Initiative for Chronic Obstructive Lung Disease (GOLD) Criteria recommends NIV as the first and standard mode of ventilation to be used in ARF secondary to AECOPD. NIV improves gas exchange, decreases respiratory rate, improves acute respiratory acidosis [3], reduces respiratory work load, avoids endotracheal intubation and invasive mechanical ventilation, decreases hospital length of stay, and improves survival [4–7]. However, NIV may face failure under certain real-life clinical circumstances. Patients may not tolerate the ventilatory machine well because of several factors [8], such as worsening respiratory symptoms, discomfort related to the positive-pressure ventilation, claustrophobia related to the tight-fitting mask, airway secretions, or patient–ventilator interactions [9].

Recent evidence demonstrated that high-flow nasal cannula (HFNC) therapy has been playing an increasing role in patients with hypercapnic respiratory failure [8, 10–12]. HFNC delivers a heated (37 °C) and humidified (44 mg H_2_O/L) air-oxygen mixture to patients, with a stable fraction of inspired oxygen (FiO_2_). Compared to conventional oxygen therapy, HFNC FiO_2_ can range from 21 to 100% oxygen with a flow rate up to 60 L/min through a special nasal cannula. The high flow rate creates a positive end-expiratory pressure (PEEP) effect that may help to counterbalance intrinsic PEEP [13], reducing inspiratory resistance, providing adequate airflow, warm gases, and enhanced respiratory tract mucus clearance [9]. HFNC also washes out the anatomical dead space of the upper airways, facilitating carbon dioxide removal [13–15].

### Objectives {7}

Based on those potential mechanisms above, it is reasonable to use HFNC in AECOPD patients [1], but its efficacy and safety in such patients are not clear [16]. In order to unite the advantages of NIV and HFNC, we designed this trial to evaluate the effect of HFNC with sequential NIV versus NIV alone on patients with mild-to-moderate AECOPD.

## Materials and methods: participants, interventions, and outcomes

### Trial design and study setting {8&9}

**Fig. 1:**
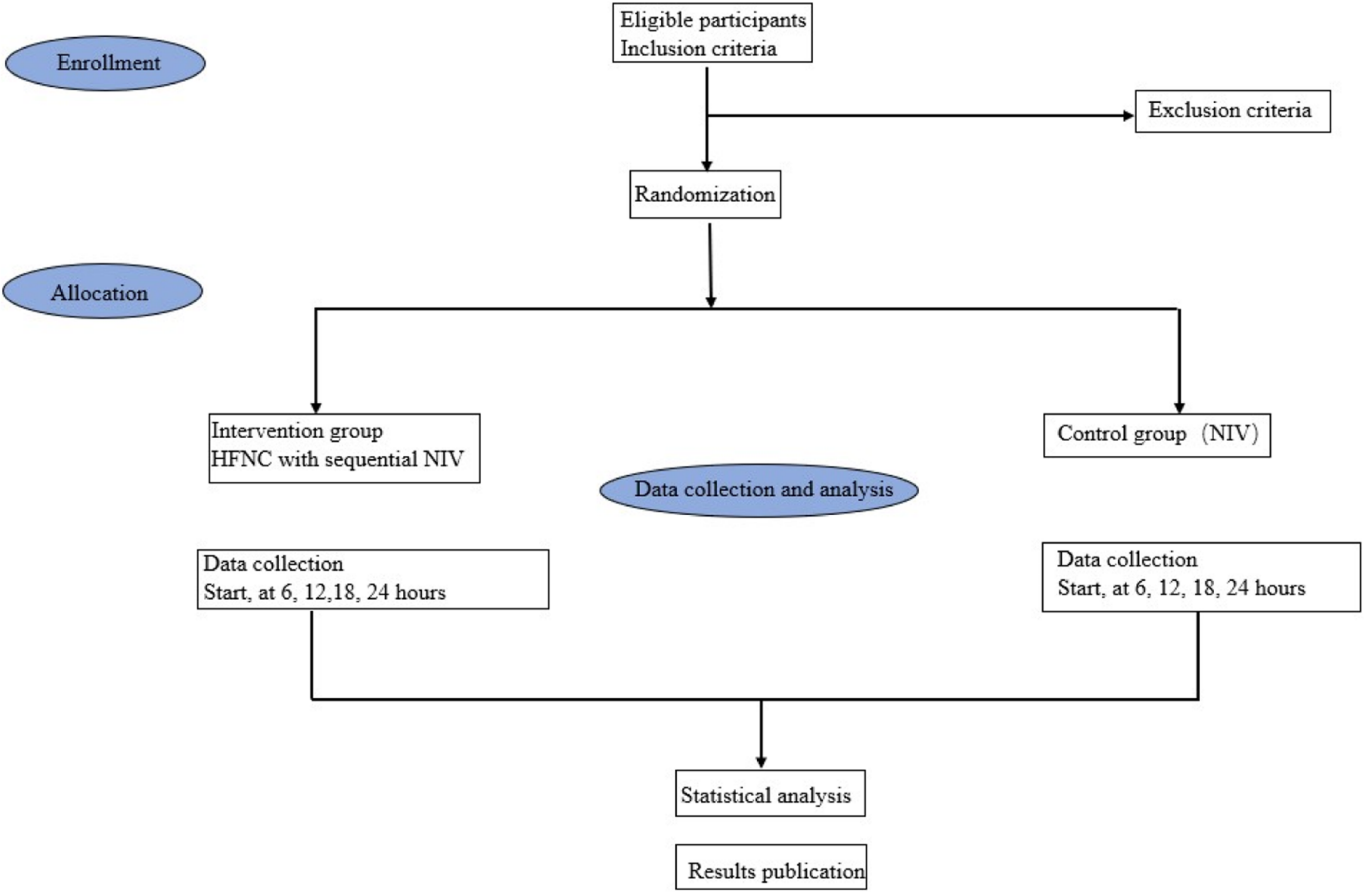
Clinical trial flow chart

This is an investigator-initiated, unblinded, single center, randomized controlled trial. This trial will be conducted in the emergency department, emergency intensive care unit, and respiratory intensive care unit of a large, urban, tertiary-care, teaching hospital. Written informed consent will be gained from all patients or their legal representatives. The flow chart of the clinical trial will be shown as (Fig. 1).

### Participants and eligibility criteria {10}

We will enroll adult patients of any gender who are 18 years old or older with a diagnosis of mild-to-moderate AECOPD according to the most recent GOLD criteria. Mild-to-moderate AECOPD is currently defined as an acute hypercapnic respiratory failure with an arterial pH between 7.25 and 7.35 and a PaCO_2_ ≥ 55 mmHg [4, 17, 18].

Exclusion criteria are as follows: (1) patients on home NIV; (2) those who have received HFNC or NIV prior to this study enrollment; (3) those who have unstable clinical conditions, such as the need for vasopressors to maintain blood pressure, acute coronary syndrome, life-threatening arrhythmias, cardiac or respiratory arrest requiring cardiopulmonary resuscitation or tracheal intubation; (4) those who refuse informed consent; (5) those who are agitated or have altered mental status characterized by a Richmond Agitation Sedation Scale (RASS, scores between − 5 and + 4, the higher the score is, the more agitated the patient is (unarousable, deep sedation, moderate sedation, light sedation, drowsy, alert and calm, restless, agitated, very agitated, combative)) ≥ 2 [17]; (6) those who are diagnosed with a failure in two or more than two organs; (7) those who have recent facial or neck trauma, congenital or pre-existing facial or neck deformities or nausea, vomiting, or hematemesis which may influence the proper application of HFNC or NIV; and (8) pregnancy [19].

### Who will take informed consent? {26a}

Written informed consent will be collected from all trial participants or their authorized surrogates. A researcher from the research team, who is trained with the study protocol will confirm the patient’s willingness to participate and eligibility criteria and obtain an informed consent.

### Interventions

#### Intervention description {11a}

In the intervention group, patients will receive HFNC when they are awake in daytime and early evening hours. When they are asleep or at night between 10 pm and 6 am, these patients will change to NIV. The HFNC group will be given a flow rate of 60 L/min and a temperature of 37° C [17]. If a patient is not tolerating these settings, flow rate and temperature will be titrated to the maximum tolerated level, but with a minimum flow rate of 30 L/min.

#### Explanation for the choice of comparators {6b}

In the control group as well as during the NIV period of the intervention group, patients will receive NIV through full or nasal mask with any available ventilator in the hospital. The ventilator will be set to Pressure Support Ventilation (PSV) mode according to usual practice, including a maximal tolerated inspiratory pressure to obtain an expired tidal volume of 6–8 mL·kg^−1^ of ideal body weight and a PEEP between 3 and 5 cm H_2_O^17^.

The FiO_2_ will be set for both groups to maintain a peripheral oxygen saturation (SpO_2_) of 88–92% [4]. Besides the ventilatory support listed above, other standard therapies will be given based on current GOLD guidelines.

#### Criteria for discontinuing the trial {11b}

Participants are allowed to discontinue and end their participation in the trial at any time for any reason. Each patient’s attending physician will make the decision to cease HFNC or NIV at any time when encountering emergency circumstances and/or for any urgent medical reasons if the patient shows signs of worsening respiratory failure or if they show one or more of the following conditions: respiratory pauses, with a loss of consciousness defined as GCS score less than 8/15 [17], bradycardia (heart rate < 50 beats per minute (bpm)) and tachy-arrythmia (heart rate > 150 bpm) [20], hypotension with a blood pressure < 90/60 mmHg [11], psychomotor agitation making nursing care impossible or requiring sedation [2], and respiratory or cardiopulmonary arrest. If such conditions happen to the patient, they may need to be intubated either to protect the airway or to manage tracheal secretions and given mechanical ventilation as per usual practice. Finally, the trial will end if the patient or legal representative rescinds their informed consent.

#### Provisions for post-trial care {30}

We expect that intervention-related severe adverse events in this trial will be unlikely because the use of HFNC and NIV are both ventilatory support in patients with respiratory failure in current practice and we will upgrade to NIV or intubation if necessary. Nevertheless, we plan to provide health care and waive the fees about ventilation for participants who may encounter trial procedure-related medical situations. Post-trial care will not be provided.

#### Outcomes {12}

The primary endpoint is the mean difference in PaCO_2_ from baseline to 24 h after randomization. Secondary endpoints include the following: (1) the mean difference in PaCO_2_ from baseline to 6, 12, and 18 h after randomization; (2) dyspnea score at 6, 12, 18, and 24 h; (3) overall discomfort score related to the ventilatory strategy and proportion of patients showing poor tolerance to treatment at 24 h (e.g., complaining about noise, pressure, temperature, gastric distension, vomiting, or claustrophobia); (4) the proportion of patients who change treatment strategies (switch to the other ventilatory strategy or to no support); (5) rate of treatment failure, defined as the proportion of patients who had PaCO_2_ worsening or reduction < 10 mmHg from baseline, or worsening with no improvement of the dyspnea or respiratory rate > 30 breaths per minute; (6) respiratory rate; (7) rate of endotracheal intubation within 24 h; (8) length of hospital stay; and (9) all-cause mortality.

#### Sample size calculation {14}

Based on previous studies [21], we set the non-inferiority cutoff at 5 mmHg. So, we assume that in the intervention group, PaCO_2_ may not elevate more than 5 mmHg after 24 h of the high-flow nasal cannula therapy with sequential noninvasive ventilation. According to previous studies, we also assume that the PaCO_2_ of intervention group is around 54 mmHg, and the control group is around 55 mmHg. Standard deviation is 9 mmHg, after 1:1 ratio randomization, and to assess non-inferiority using an *α* = 0.05, power = 0.80, and 1-side testing, 58 subjects were needed totally. Allowing for 10% loss to follow-up, we assume that we will need to recruit 70 patients. However, if loss to follow-up is greater than 10%, we aim to continue recruitment until 58 patients have a primary outcome recorded.

### Assignment of interventions, allocation and concealment mechanisms {16}

Patients will be randomized into either an intervention group or a control group through a 1:1 ratio using a computer-generated randomization sequence via an independent investigator, who will not be involved in the trial. Allocation concealment will be maintained using sequentially numbered sealed opaque envelopes. Each envelope contains the patient’s allocation to either control or intervention group, with a unique study patient code. An independent research assistant is responsible for keeping these envelopes unopened and allocated. The baseline is defined as the time of randomization.

### Assignment of interventions: blinding {17}

Because of the design of this trial using HFNC or NIV, neither the investigators nor the patients can be blinded to the treatment allocation.

### Recruitment {15}

We will recruit participants from the emergency department, emergency intensive care unit and respiratory intensive care unit of a large, urban, tertiary-care, teaching hospital. Investigators will explain the purpose, methods, possible risks, benefits, and rights to patients who are eligible to participant in this trial. If the participant agrees to be enrolled and meets the eligibility criteria, the investigator will ask the patient or their guardian to sign the study’s informed consent form.

### Participant retention and withdrawal

Once the participants are enrolled, the research team has the responsibility to clarify the goal and obligation to the participants in order to achieve a low rate of loss to intervention. Before the start of the intervention, investigators will take time to educate and explain the duration of the intervention and possible adverse effects to all patients and their legal representatives in details. All patients will be informed that they have the right to withdraw from the study at any time during the intervention and the Data Monitoring Committee (DMC) will discuss and analyze the reasons for dropouts with data collectors, which will be documented in a standardized form. All efforts will be made to complete the follow-up and data collection and avoid missing data. Analyses will be conducted in complete cases. We will also perform sensitivity analyses by imputing the missing data to worst cases.

The data and safety monitoring committee (DSMC) will consist of four qualified members from the Peking Union Medical College Hospital, including at least one statistician and intensive care or respiratory expert independent from the site trial staff. The DSMC is independent from the trial researchers, blinded to the assignment, and will monitor the progress of the trial and safety issues and require data for analyses.

### Data collection and measurement {18}

We will collect patient personal and baseline clinical characteristics as delineated above (e.g., age, gender, RASS, etc.). Furthermore, we will collect vital signs, systolic and diastolic pressure, heart rate, peripheral oxygen saturation, the presence of dyspnea based on the Borg dyspnea scale (score from 0 to 10 after 6 min’ walk, 0 means no dyspnea, 10 means the maximum), the arterial blood gas at inclusion, and starting time and settings for HFNC/NIV at 6, 12, 18, and 24 h. During the study intervention, patients will have continuous SpO_2_, electrocardiogram, respiratory rate, and noninvasive blood pressure monitoring. All relevant variables and endpoints will be evaluated at 6, 12, 18, and 24 h after randomization. In particular, we will record the NIV and HFNC settings as well as the proportion of patients who change treatment modality during the study period (i.e., switch to the other ventilatory strategy or to no ventilatory support). The discomfort score related to different ventilatory strategies will also be assessed at the established time points, and the proportion of patients showing poor tolerance to the treatment but who do not interrupt or withdrawal due to noise, temperature of flow, gastric distension, vomiting, or claustrophobia will be recorded. We will also record the rate of treatment failure due to a worsening PaCO_2_, a less than 10 mmHg reduction in PaCO_2_ from baseline, a worsening or lack of improvement in dyspnea, or a respiratory rate > 30 breaths per minute. Hospital length of stay and any-cause in-hospital mortality will all be collected. The main schedule of enrolment, intervention, and assessment were listed in the Table 1.

**Table 1:** Schedule of enrolment, intervention, and assessment

Time point	Study period						
	Enrollment	Allocation	Post-allocation				
			Start	6 h	12 h	18 h	24 h
Enrollment							
Eligibility	** × **						
Informed consent	** × **						
Allocation		** × **					
Intervention							
Intervention group			** × **	** × **	** × **	** × **	** × **
Control group			** × **	** × **	** × **	** × **	** × **
Assessment							
Patient characteristics	** × **						
Vital signs	** × **		** × **	** × **	** × **	** × **	** × **
Medical history	** × **						
Laboratory results							
ABG			** × **	** × **	** × **	** × **	** × **
RASS score			** × **	** × **	** × **	** × **	** × **
Borg Dyspnea score			** × **				** × **
Discomfort score			** × **				** × **
Secretions							
Treatment change							** × **
Treatment failure							** × **
Endotracheal intubation							** × **

### Methods in analysis to handle protocol non-adherence and any statistical methods to handle missing data {20c}

All efforts will be made to complete the follow-up and data collection and avoid missing data. Analyses will be conducted in complete cases. We will also perform sensitivity analyses by imputing the missing data to worst cases.

### Data management {19}

All collected data will be stored and managed using a web-based data management system. Researchers are responsible for the data collection, especially for checking that all data related to this trial are filled out in the data manage system correctly and completely. A data management plan will be created beforehand for data validation.

### Confidentiality {27}

Personal data of the participants will be treated as confidential and stored securely during the whole course of this trial.

### Statistical analysis {20}

The primary and secondary endpoints will be analyzed according to the intention-to-treat principle. We will use descriptive statistical methods to measure patient characteristics and baseline clinical features. For primary and secondary outcomes, continuous variables will be presented as means ± standard deviations or median interquartile ranges (IQR). Categorical variables will be expressed as counts with percentages. We will use Student’s *T* tests or Mann–Whitney *U* tests to evaluate the differences between treatments in continuous variables according to normal distribution. Categorical variables will be compared using chi-squared or Fisher’s exact tests. All data collected will be analyzed by an independent statistical expert using SPSS (SPSS 22.0). A *p*-value < 0.05 will be considered statistically significant.

### Oversight and monitoring

#### Composition of the coordinating center and trial steering committee {5d}

The coordinating center is the Peking Union Medical College Hospital. The trial steering committee consists of principal investigators responsible for interventions. Other clinicians especially the residents in every ward will be responsible for follow-up, data collection for the trial.

#### Adverse event reporting and harms {22}

Adverse events reported by the participants or noticed by the clinicians or the caring nurses will be reported directly to the DSMC and recorded in the digital case report form and data management system.

#### Frequency and plans for auditing trial conduct {23}

Audit reports will be given by the trained auditors at three different time points, namely after the first participant is enrolled, at 12 months after launching the trial, and before the trial is closed out. Eligibility of recruited participants, collection of consent, and data integrity will be checked at each study site.

## Discussion

In the past few years, there have been multiple studies investigating the application of NIV and HFNC in AECOPD patients, but a consensus has remained elusive. Previous studies have shown that HFNC has several properties superior to NIV in AECOPD patients, such as better patient tolerance and improved patient–ventilator interaction. Nevertheless, NIV has a longer clinical track-record with high quality evidence supporting its position as the gold-standard respiratory support method for managing AECOPD patients.

The recent systematic review from Pisani et al. reported that HFNC has been used successfully in AECOPD patients as an alternative to NIV due to its reduction in work of breathing and comfort [1]. Another recent ultrasound study examined diaphragm displacement and diaphragm thickening fraction in a cross-over study including 30 AECOPD patients with hypercapnic acute respiratory failure receiving NIV for more than 24 h [20]. That study found that the diaphragm thickening fraction in the HFNC group remained unchanged which implied equivalent clinical efficacy to NIV, while improving patient comfort [12]. A single-center randomized controlled cross-over study concluded that treatment with NIV led to a greater reduction in PaCO_2_ than treatment with HFNC, but the latter was superior across a range of tolerability aspects [9, 22]. In a short-term crossover clinical trial, Rezaei et al. found no difference in any parameters between AECOPD patients treated with NIV or HFNC (e.g., respiratory rate and PaCO_2_), however, HFNC appeared better in patients with AECOPD to reduce dyspnea scores and improve respiratory distress [23]. Li et al. conducted a prospective, randomized, controlled trial which enrolled 320 patients with AECOPD who were given either HFNC or conventional oxygen therapy, finding that the PaCO_2_ of the HFNC group was lower at 24 h and these patients had improved prognoses [24]. Xu-Yan Li et al. conducted a prospective randomized controlled study comparing the effectiveness of HFNC to conventional oxygen therapy in AECOPD patients, and found HFNC had improved the treatment failure [24]. However, other prospective study compared the effect of HFNC to NIV in hospitalized severe AECOPD patients and found no significant differences in 6 and 24 h ABG analyses, as well as 30-day intubation and mortality rates [25]. HFNC has been associated with fewer complications, better tolerance, and less naso-facial skin breakdown than NIV [15]. Most significantly, Cortegiani et al. performed a multicenter, non-inferiority randomized trial comparing HFNC to NIV in nine centers in Italy. The patients they randomized to the HFNC group had worsening oxygenation during the first six hours. Subsequently, 32% of these patients underwent NIV during their hospitalization, but they also had a longer length of NIV than those originally allocated to the NIV group [17]. These findings suggest that HFNC and NIV may play a complementary role in AECOPD treatment.

Above all, no matter the clinical parameters or prognosis, HFNC may be noninferior to NIV. HFNC shows advantages in better patient comfort and tolerance, fewer complications, improved warming and humidification of their respiratory tracts, and better sputum excretion. However, if AECOPD patients are asleep, HFNC may increase the risk of CO_2_ retention and respiratory acidosis. On the other hand, NIV may face failure because of discomfort related to the ventilation and the mask, decreased secretion clearance, and patient–ventilator interaction mismatch.

This study aims to try to take advantage of both the strengths of HFNC and NIV to improve clinical care for AECOPD patients in the ED and critical care environments. We believe that the work of this trial will help us better understand whether HFNC with sequential NIV has any advantages over NIV alone. If this ventilatory strategy proves beneficial or non-inferior, it could provide crucial clinical evidence for improving care for AECOPD patients.

## Trial status

Participant enrollment will be started on 1 November 2022.The completion is expected to be finished on 31 January 2025.

## Data Availability

After this study is completed, the final trial data and statistical codes will be available from the corresponding authors upon reasonable request, except for participants’ personal information. The results will be published in peer reviewed journals. Findings will be shared with the participants, healthcare workers, the general public, and relevant departments through open-access articles, public talks, conferences, and final reports.

## Abbreviations

COPD: Chronic obstructive pulmonary disease
HFNC: High-flow nasal cannula
NIV: Noninvasive ventilation
AECOPD: Acute exacerbation of chronic obstructive pulmonary disease
GOLD: Global Initiative for Chronic Obstructive Lung Disease
FiO_2_: Inspired fraction of oxygen
PEEP: Positive end-expiratory pressure
PaCO_2_: Partial pressure of arterial carbon dioxide
